# Split Versus Single Applications of Solid and Liquid Vermicompost: Impacts on Growth, Chlorophyll Content and Soil Characteristics of Tea (*Camellia sinensis* L.) Seedlings

**DOI:** 10.3390/plants15203082

**Published:** 2026-10-09

**Authors:** Türker Oğuztürk, Turan Yüksek, Filiz Yüksek, Merve Sipahi, Bahriye Pirim, Rabia Erkmen, Gülcay Ercan Oğuztürk

**Affiliations:** 1 Department of Landscape Architecture, Faculty of Engineering and Architecture, Recep Tayyip Erdoğan University, Rize 53020, Türkiye; turker.oguzturk@erdogan.edu.tr (T.O.); turan.yuksek@erdogan.edu.tr (T.Y.); bahriye_pirim20@erdogan.edu.tr (B.P.); rabia_erkmen22@erdogan.edu.tr (R.E.); 2 Rize Forest Enterprise Directorate, Trabzon Regional Directorate of Forestry, General Directorate of Forestry, Rize 53020, Türkiye; filiz6108@gmail.com; 3 Department of Interior Architecture and Environmental Design, Faculty of Architecture, Design and Fine Arts, Osmaniye Korkut Ata University, Osmaniye 80000, Türkiye; mervesipahi@osmaniye.edu.tr

**Keywords:** growth performance, soil properties, split application, tea seedlings, vermicompost

## Abstract

Limited information is available on how vermicompost form, form-specific dose, and application schedule jointly affect tea seedling development. This 135-day humid outdoor nursery study evaluated solid and liquid vermicompost applied at two doses as either single or three split applications to 270 individually potted *Camellia sinensis* seedlings assigned to eight factorial treatments and an untreated control. Repeated measurements of seedling height, root collar diameter, and leaf chlorophyll content were analyzed using linear mixed-effects models fitted by restricted maximum likelihood (REML); the control was retained only in supplementary one-way ANOVAs. Soil pH, electrical conductivity (EC), and temperature were analyzed by two-way ANOVA. Vermicompost form, measurement time, and their interaction significantly affected seedling height and root collar diameter. Application schedule significantly affected root collar diameter (F = 7.35, *p* = 0.007) and chlorophyll content (F = 19.74, *p* < 0.001), but not seedling height, whereas dose within form showed no significant main effect on any of these traits. The form × application schedule × time interaction was significant for seedling height, whereas dose within form × application schedule × time was significant for root collar diameter and chlorophyll content. Treatment differences generally emerged from day 30 onward. The 30 g solid vermicompost treatment applied in three equal doses produced the highest final seedling height (24.80 cm) and root collar diameter (14.00 mm) and the highest chlorophyll content recorded during the experiment (914.98 µmol m^−2^ on day 75). Treatment, measurement time, and their interaction significantly affected soil pH, EC, and temperature; pH remained within 4.90–5.76, and EC values did not indicate salinity stress. Selected split applications of solid vermicompost therefore showed potential to improve tea seedling growth, but significant higher-order interactions demonstrated that responses depended on vermicompost form, dose within form, application schedule, trait, and measurement time.

## 1. Introduction

Tea, produced from the leaves of *Camellia sinensis* (L.) Kuntze, is an economically and culturally important crop and one of the most widely consumed beverages worldwide, second only to water [1,2]. Its high concentrations of phenolic compounds, catechins, and other bioactive constituents have generated substantial scientific and commercial interest [3,4]. The global tea sector provides employment for approximately 13 million people [5], and its market value is projected to reach USD 98.2 billion by 2033 [6]. In Türkiye, tea is cultivated predominantly in the Eastern Black Sea Region, particularly in Rize Province, where it plays a strategic role in the regional economy and rural livelihoods [7].

Tea cultivation in Türkiye began in 1938 [8,9], and the cultivated area has expanded considerably over time. However, a significant portion of the existing tea plantations has reached an advanced age, resulting in declines in yield and quality [10]. In tea plantations with declining productivity, chemical fertilizers are often applied excessively or without adequate soil-based planning to increase production. Such practices may cause important environmental problems, including the contamination of groundwater and surface water resources, deterioration of soil structure and health, and loss of biodiversity [11]. When plant nutrition practices rely primarily on producer experience rather than soil analysis, these environmental risks may be further intensified [12,13]. Another challenge is the need to adapt aging tea plantations to changing climatic conditions, including changes in temperature and precipitation and increased drought risk. In addition, the supply of high-quality tea seedlings for renewing aging plantations and establishing new plantations in the Eastern Black Sea Region remains limited. Therefore, producing high-quality tea seedlings with strong growth performance and improved tolerance to diseases, pests, and climate-related stresses is of great importance. This would support both the renewal of existing plantations and the development of sustainable tea production systems.

Considering the environmental risks associated with chemical fertilizer use, the development and wider adoption of environmentally friendly organic fertilizers that support soil and plant health are important for sustainable agriculture and are consistent with the United Nations Sustainable Development Goals. Vermicompost is among the organic amendments that have been widely investigated and used in sustainable crop production systems. Its potential for production and application at different agricultural scales has further increased interest in this fertilizer. In addition, vermicompost contains diverse microbial communities [14,15,16,17,18] and a wide range of nutrients [11,19,20,21], which can improve soil physicochemical properties and biological functioning [22,23,24,25,26], thereby creating favorable conditions for plant growth. In particular, liquid vermicompost contains dissolved organic carbon, beneficial microorganisms, and plant growth-promoting bioactive compounds. It has been reported to support root development [27,28], enhance chlorophyll accumulation, and improve plant tolerance to environmental stresses [16,29].

In tea planting material production, organic manures and bio-inoculants were evaluated, and a treatment combining vermicompost with a biofertilizer mixture produced superior performance compared with the other tested treatments and the control [30]. However, information on vermicompost application specifically in tea seedlings remains limited, particularly regarding the comparative effects of vermicompost form, dose, and application timing. Although many studies have reported beneficial effects of vermicompost on plant growth and soil properties, much of the existing research has focused on total application dose rather than application schedule. In regions with high rainfall, however, the effectiveness of organic fertilizers may depend not only on the total amount applied but also on how that amount is distributed over time. Applying the total dose at once may increase nutrient losses through leaching or result in a short-lived nutrient supply, whereas split applications may provide more sustained nutrient availability and better alignment with plant demand. Nevertheless, direct comparisons of single and split applications of solid and liquid vermicompost in tea seedlings remain scarce.

The aim of this study was to evaluate the effects of vermicompost form, dose within form, and application schedule on the growth, leaf chlorophyll content, and selected growing-medium properties of tea (*Camellia sinensis*) seedlings. The specific objectives were:

- To compare the effects of solid and liquid vermicompost on tea seedling growth;
- To determine the individual and interactive effects of dose within form and application schedule on seedling height, root collar diameter, and leaf chlorophyll content over time;
- To evaluate changes in selected growing-medium properties, including pH, electrical conductivity, and temperature, following vermicompost application;
- To identify promising vermicompost form–dose–schedule combinations that may inform sustainable fertilization strategies for tea seedling production.

By addressing these objectives, the study provides evidence to support more sustainable and environmentally responsible vermicompost management in tea seedling production.

## 2. Materials and Methods

### 2.1. Plant Material and Study Area

The study was initiated on 26 April 2024 and conducted under outdoor nursery conditions in Rize Province for 135 days. The study area is located at approximately 41°01′ N latitude and 40°31′ E longitude, and the region is characterized by a humid, high-rainfall climate. One-year-old (1 + 0) *Camellia sinensis* L. seedlings produced from seeds were used as plant material. Before the experiment, healthy seedlings with similar initial seedling heights and root collar diameters were selected and assigned to the treatment groups. The solid and liquid vermicompost materials used in the study were obtained from a vermicompost production unit under standard production conditions. All vermicompost applications during the experimental period were performed using materials obtained from the same production batch. Some physicochemical properties of the solid and liquid vermicompost materials are presented in Table 1.

### 2.2. Experimental Design

The study was established using a completely randomized design (CRD). The treatment structure comprised vermicompost form (solid or liquid), dose nested within form (30 or 60 g for solid vermicompost and 30 or 60 mL for liquid vermicompost), and application schedule (single or split application). These factors yielded eight vermicompost treatment combinations. An untreated control was included for treatment-wise comparisons but was not part of the factorial structure. Each treatment comprised 30 individually potted seedlings, which were operationally arranged into six groups of five seedlings for experimental management and data recording. The assigned vermicompost treatment was applied separately to each potted seedling; therefore, each seedling constituted an independent experimental unit. Accordingly, each treatment comprised 30 experimental units, resulting in a total of 270 seedlings across the nine treatments. The experiment was conducted under outdoor conditions in polyethylene growing bags for 135 days. When filled with the growing substrate, the polyethylene bags were approximately 19 cm in height and 8 cm in upper opening diameter, with the substrate surface maintained approximately 3.0 ± 0.5 cm below the upper rim of the bag. In split applications, the total amount assigned to each seedling was divided into three equal doses and applied at 30-day intervals within the first 90 days. In single applications, the entire amount assigned to each seedling was applied once at the beginning of the experiment. For both single and split treatments, solid vermicompost was incorporated into the growing medium of each individual bag at the time of application. Before application, liquid vermicompost was diluted to 10% (*v*/*v*) with tap water. Accordingly, the 30 and 60 mL vermicompost doses were applied as 300 and 600 mL of diluted solution per seedling, respectively; in the split treatments, these volumes were divided into three equal applications. The treatment groups and experimental design are presented in Table 2.

### 2.3. Growth Conditions and Measurement Schedule

The seedlings were grown in polyethylene growing bags containing a standard growing medium used for tea seedling production. All treatments received the same maintenance practices, and the seedlings were grown under natural environmental conditions. Measurements were conducted at 15-day intervals throughout the 135-day experimental period. Seedling height and root collar diameter were measured on day 0 and subsequently at 15-day intervals through day 135, resulting in ten measurement occasions. Leaf chlorophyll content and growing-medium properties (pH, electrical conductivity, and temperature) were measured from day 15 through day 135, resulting in nine measurement occasions. Different and independent growing-medium samples were evaluated at each measurement occasion.

### 2.4. Growth, Physiological, and Growing-Medium Measurements

Root collar diameter was measured using a digital caliper to the nearest 0.01 mm. Seedling height was measured from the surface of the growing medium to the shoot tip (apical meristem) using a ruler. Leaf chlorophyll content was measured using an Apogee MC-100 chlorophyll meter (Apogee Instruments Inc., Logan, UT, USA). At each measurement occasion, chlorophyll content was measured on one leaf per seedling, and the resulting value was used as the chlorophyll observation for that seedling. Growing-medium pH and electrical conductivity (EC) were measured using a portable Hanna Instruments meter (Hanna Instruments, Temse, Belgium), with EC values recorded in mS cm^−1^ as indicated by the instrument. Growing-medium temperature was measured concurrently with pH and EC at each measurement occasion. Growing-medium temperature was measured concurrently with pH and EC at each measurement occasion.

### 2.5. Statistical Analysis

Data were analyzed using IBM SPSS Statistics, version 23.0 (IBM Corp., Armonk, NY, USA). Repeated measurements of seedling height, root collar diameter, and leaf chlorophyll content were primarily analyzed using linear mixed-effects models fitted by restricted maximum likelihood (REML). Separate one-way analyses of variance (ANOVA) conducted at each measurement occasion were used as supplementary treatment-wise comparisons. Vermicompost form, dose nested within form, application schedule, measurement time, and their interactions were specified as fixed effects. Measurement occasion was treated as a categorical fixed effect, whereas numeric time was included as a random slope. Seedling identity was specified as the subject, and a random intercept and random linear time slope were fitted using an unstructured covariance matrix. For seedling height and root collar diameter, measurements obtained from days 15–135 were included as repeated responses, and the corresponding day-0 measurement was included as a covariate. No baseline covariate was included in the chlorophyll model because chlorophyll was not measured on day 0. Type III tests of fixed effects were used. Significant higher-order interactions were interpreted using estimated marginal means. The factorial mixed-model analyses included the eight vermicompost treatment combinations; the untreated control was retained in the supplementary treatment-wise analyses but excluded from the factorial models because vermicompost form, dose, and application schedule were not defined for the control. Because different and independent growing-medium samples were evaluated at each measurement occasion, growing-medium properties (pH, EC, and temperature) were analyzed using two-way ANOVA to test the main effects of treatment, time, and their interaction. For the ANOVA-based analyses, normality of data distribution was assessed using skewness and kurtosis values, and homogeneity of variances was evaluated using Levene’s test. When significant differences were detected in the ANOVA-based analyses, means were compared using Tukey’s HSD test. Statistical significance was set at *p* ≤ 0.05.

## 3. Results

### 3.1. Root Collar Diameter

The effects of vermicompost treatments on root collar diameter were not statistically significant on days 0 and 15 (*p* = 0.515 and *p* = 0.429, respectively), but became significant from day 30 onward (all *p* < 0.001; Table 3).

No statistically significant differences were observed among treatments at the beginning of the experiment or on day 15. On day 30, the highest mean root collar diameter was recorded in T5 (30 g solid vermicompost applied once; 9.54 mm), whereas the lowest value was recorded in T3 (60 mL liquid vermicompost applied once; 7.67 mm). By day 135, the highest mean values were recorded in T6 (30 g solid vermicompost applied in split doses; 14.00 mm) and T8 (60 g solid vermicompost applied in split doses; 13.96 mm). These treatments were followed by T5 and T7, with mean values of 12.75 and 12.55 mm, respectively. Among the liquid vermicompost treatments, the highest final mean was recorded in T4 (60 mL applied in split doses; 10.81 mm). Overall, the final root collar diameter values were higher under solid vermicompost, particularly with split application, than under liquid vermicompost or the untreated control (Table 3).

### 3.2. Seedling Height

The effects of vermicompost treatments on seedling height were not statistically significant on days 0 and 15 (*p* = 0.509 and *p* = 0.069, respectively), but became significant on day 30 (*p* = 0.015) and remained significant at all subsequent measurement occasions (all *p* <0.001; Table 4). On day 30, the highest mean seedling height was recorded in T3 (60 mL liquid vermicompost applied once; 14.33 cm), whereas the lowest value was recorded in T7 (60 g solid vermicompost applied once; 12.32 cm). At the end of the experiment, the highest mean seedling height was recorded in T6 (30 g solid vermicompost applied in split doses; 24.80 cm), followed by T8 (60 g solid vermicompost applied in split doses; 24.26 cm). The lowest final value was recorded in T1 (30 mL liquid vermicompost applied once; 16.47 cm). Relative to its day-0 value, seedling height in T6 increased by 110.17%. At day 135, all solid vermicompost treatments produced significantly greater seedling heights than the untreated control and the liquid vermicompost treatments. Although seedling height also increased under liquid vermicompost, the final values remained lower than those obtained under solid vermicompost (Table 4). Between day 0 and day 135, the relative increase in seedling height ranged from 35.22% in T1 to 110.17% in T6. The next highest increases were observed in T8 (94.39%) and T7 (79.95%), whereas the untreated control showed an increase of 41.10% (Table 5).

### 3.3. Leaf Chlorophyll Content

Vermicompost treatments had no significant effect on leaf chlorophyll content at the first chlorophyll measurement on day 15 (*p* = 0.514). Significant treatment differences were observed from day 30 to day 90 and again on days 120 and 135 (all *p* < 0.001; Table 6). Treatment rankings varied among measurement occasions, indicating distinct temporal response patterns rather than the consistent superiority of a particular vermicompost form or treatment. The highest chlorophyll content recorded during the experiment was 914.98 µmol m^−2^ in T6 (30 g solid vermicompost applied in split doses) on day 75. At the final measurement, the highest value was recorded in T4 (60 mL liquid vermicompost applied in split doses; 825.35 µmol m^−2^; Table 6). Between days 15 and 135, T4 also showed the largest absolute and relative increases (632.86 µmol m^−2^ and 328.78%, respectively), followed by T6 with a relative increase of 309.30% (Table 7).

### 3.4. Soil Properties

**Figure 1:**
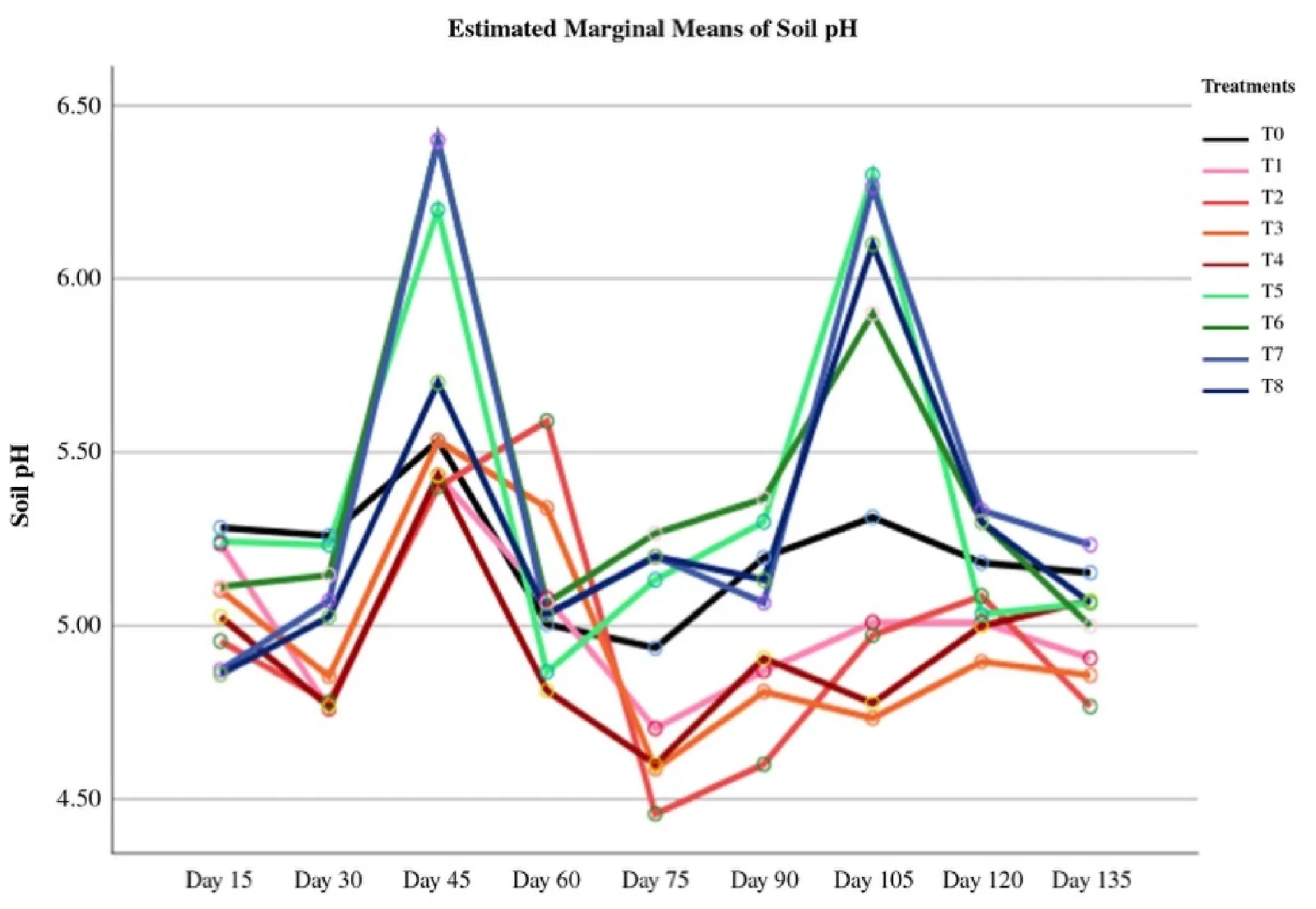
Estimated marginal means of soil pH across measurement days under different vermicompost treatments.

**Figure 2:**
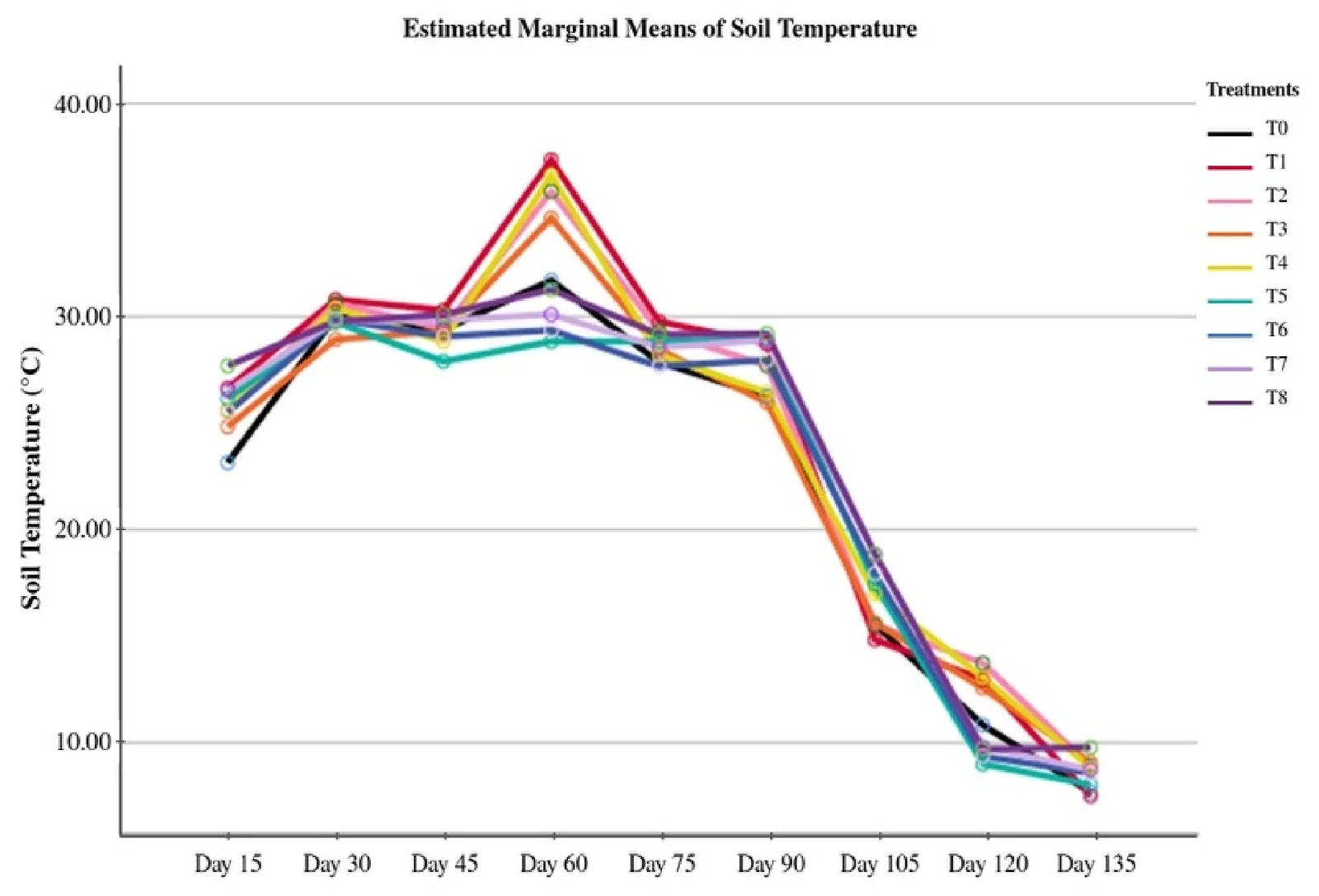
Effects of vermicompost treatments on soil temperature across measurement times.

**Figure 3:**
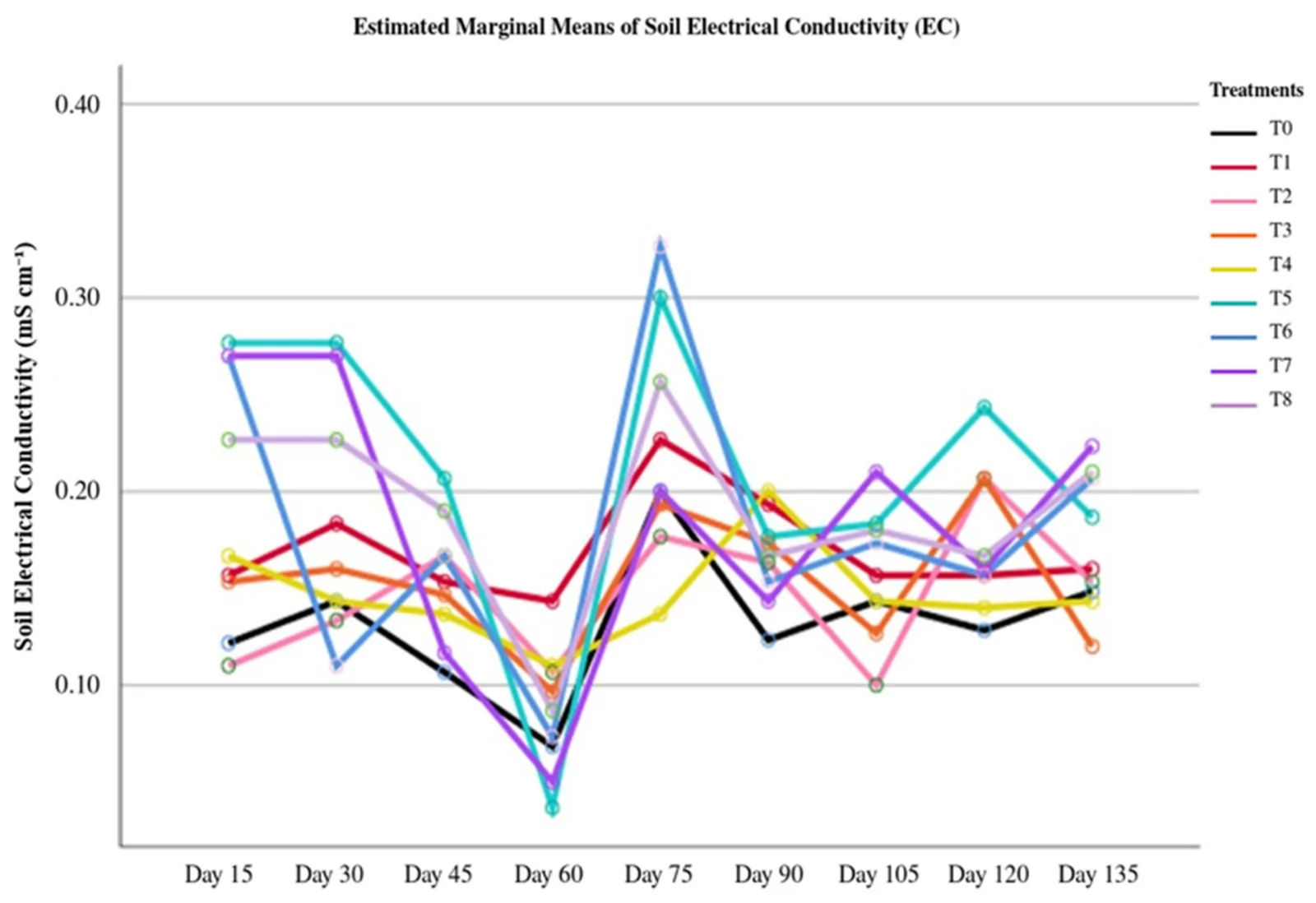
Effects of vermicompost treatments on soil electrical conductivity (EC; mS cm^−1^) across measurement times.

Two-way ANOVA showed that measurement time, vermicompost treatment, and their interaction significantly affected soil pH, temperature, and electrical conductivity (EC) (all *p* ≤ 0.001; Table 8). Because the measurement time × treatment interaction was significant for all three soil properties, the marginal means and Tukey groupings presented in Table 9 were interpreted descriptively and evaluated together with the time-specific treatment patterns shown in Figure 1, Figure 2 and Figure 3. Across measurement occasions, mean pH ranged from 4.90 on day 75 to 5.76 on day 45, whereas treatment marginal means ranged from 4.93 in T4 to 5.40 in T6. Mean temperature increased from 25.49 °C on day 15 to 32.75 °C on day 60 and subsequently decreased to 8.48 °C on day 135; treatment marginal means ranged from 22.49 to 24.32 °C. Mean EC ranged from 0.08 mS cm^−1^ on day 60 to 0.22 mS cm^−1^ on day 75, with treatment marginal means ranging from 0.13 mS cm^−1^ in the untreated control to 0.21 mS cm^−1^ in T5 (Figure 1, Figure 2 and Figure 3; Table 9).

Soil temperature generally increased from 25.49 °C on day 15 to a maximum of 32.75 °C on day 60 and then decreased progressively to a minimum of 8.48 °C on day 135 (Figure 2 and Table 9). Thus, the most pronounced decline occurred during the second half of the experiment.

Electrical conductivity (EC) values ranged from 0.08 to 0.22 mS cm^−1^ during the experiment (Figure 3 and Table 9). Across measurement occasions, the highest mean EC was recorded on day 75 (0.22 mS cm^−1^), whereas the lowest was recorded on day 60 (0.08 mS cm^−1^). When averaged across measurement occasions, the highest mean EC was observed in the treatment receiving a single application of 30 g solid vermicompost (0.21 mS cm^−1^), whereas the control had the lowest mean value (0.13 mS cm^−1^; Table 9).

### 3.5. Longitudinal Mixed-Model Analysis of Seedling Growth and Leaf Chlorophyll Content

Type III tests of fixed effects from the REML-based linear mixed models for seedling height, root collar diameter, and leaf chlorophyll content are presented in Table 10.

**Figure 4:**
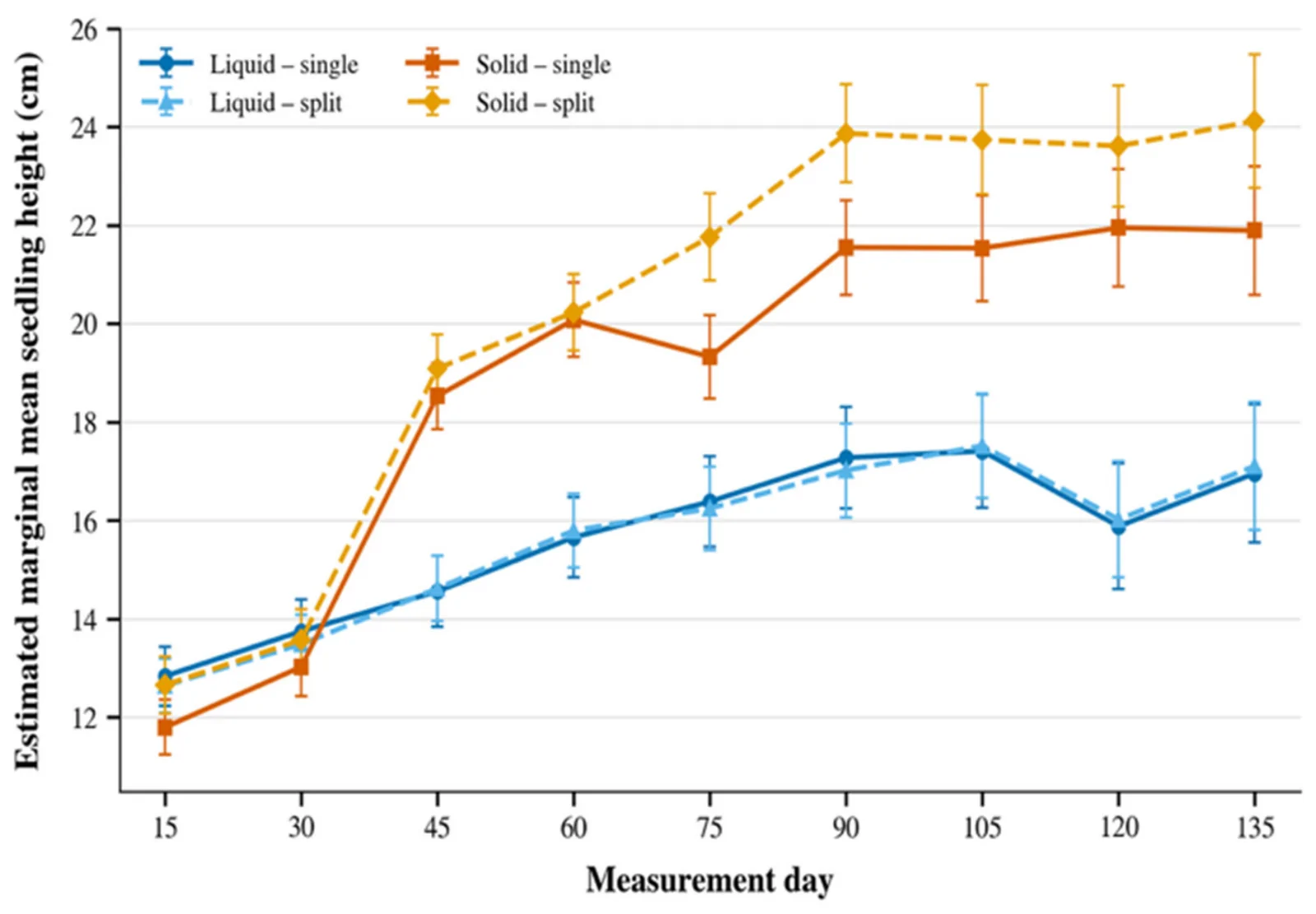
Vermicompost form × application schedule × time interaction for seedling height. Values are baseline-adjusted estimated marginal means with 95% confidence intervals.

Analysis using a linear mixed-effects model fitted by REML showed that after adjustment for baseline seedling height, seedling height was significantly affected by vermicompost form (*F* (1, 165.57) = 103.30, *p* < 0.001), time (*F* (8, 811.95) = 193.40, *p* < 0.001), and the form × time interaction (*F* (8, 811.95) = 45.69, *p* < 0.001). The form × application schedule × time interaction was also significant (*F* (8, 811.95) = 2.91, *p* = 0.003), whereas the remaining experimental effects reported in Table 10 were not significant (*p* > 0.05). As shown in Figure 4, estimated marginal means adjusted for baseline seedling height indicated that the effect of application schedule on temporal seedling growth differed between vermicompost forms. Single and split applications produced similar growth trajectories under liquid vermicompost. Under solid vermicompost, however, split application resulted in progressively greater adjusted seedling height, particularly from day 75 onward, and produced the highest adjusted mean on day 135 (24.11 cm).

**Figure 5:**
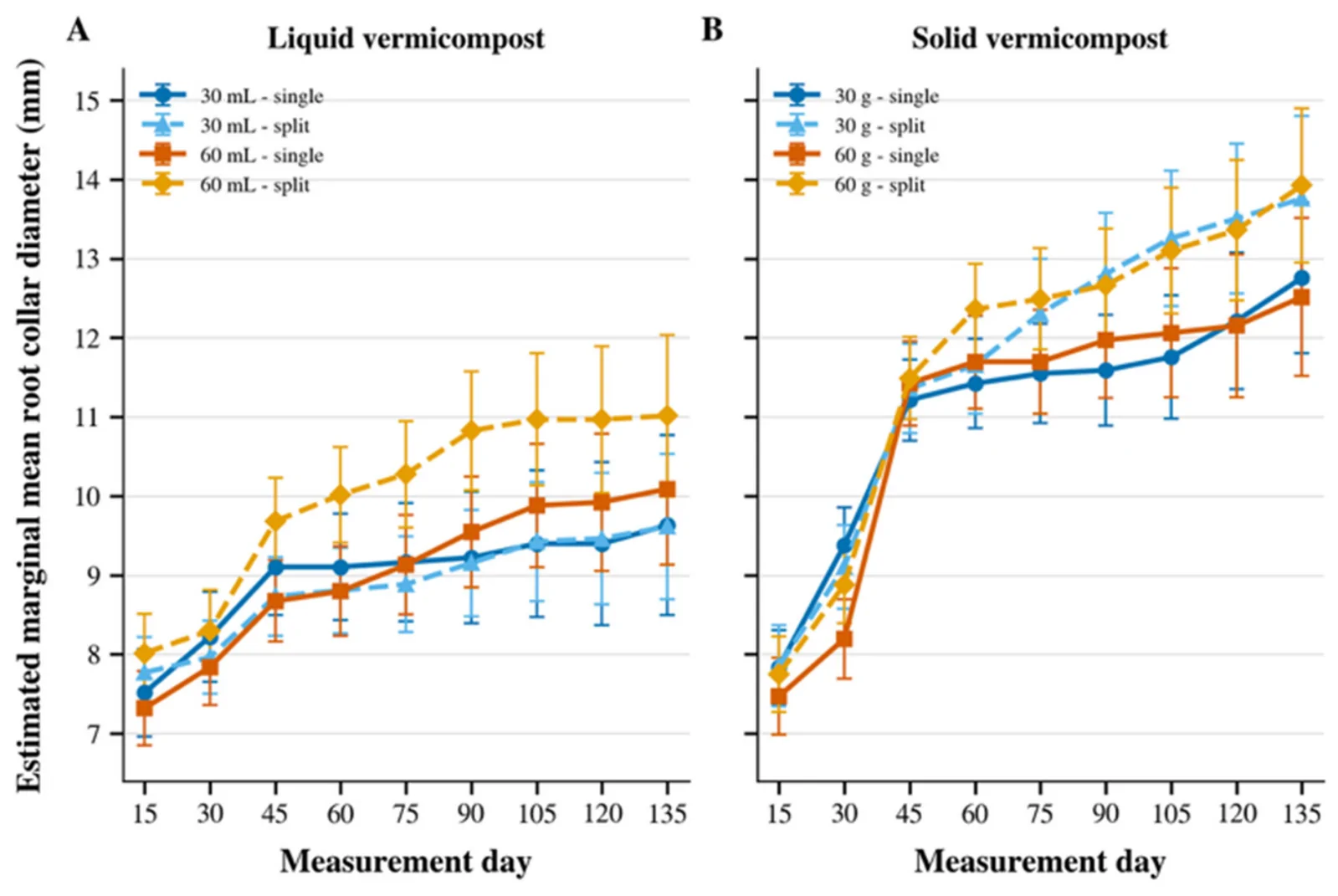
Dose within vermicompost form × application schedule × time interaction for root collar diameter. (**A**) liquid vermicompost treatments; (**B**) solid vermicompost treatments. Values are baseline-adjusted estimated marginal means with 95% confidence intervals.

After adjustment for baseline root collar diameter, vermicompost form (*F* (1, 164.88) = 105.62, *p* < 0.001), application schedule (*F* (1, 163.23) = 7.35, *p* = 0.007), time (*F* (8, 784.70) = 155.71, *p* < 0.001), and the form × time interaction (*F* (8, 784.70) = 31.94, *p* < 0.001) significantly affected the root collar diameter. The dose within form × application schedule × time interaction was also significant (*F* (8, 784.70) = 2.04, *p* = 0.039), whereas the remaining experimental effects reported in Table 10 were not significant (*p* > 0.05). As shown in Figure 5, baseline-adjusted estimated marginal means indicate that temporal changes in root collar diameter depend on dose within vermicompost form and application schedule. Treatment combinations had relatively similar adjusted means on day 15, but their trajectories diverged as the experiment progressed. On day 135, the highest adjusted mean was obtained with 60 g solid vermicompost applied in split doses (13.92 mm), followed by 30 g solid vermicompost applied in split doses (13.76 mm). Among the liquid treatments, the highest final adjusted mean was obtained with 60 mL applied in split doses (11.01 mm). At the final measurement occasion, split application was associated with greater root collar diameter, particularly under solid vermicompost.

Leaf chlorophyll content was significantly affected by application schedule (*F* (1, 166.43) = 19.74, *p* < 0.001) and time (*F* (8, 912.30) = 239.76, *p* < 0.001). The form × application schedule (*F* (1, 166.43) = 7.04, *p* = 0.009) and dose within form × application schedule interactions (*F* (1, 166.43) = 15.23, *p* < 0.001) were also significant. All interaction terms involving time were significant (*F* (8, 912.30) = 3.86–28.98, all *p* < 0.001), including the dose within form × application schedule × time interaction (*F* (8, 912.30) = 4.73, *p* < 0.001). In contrast, the main effects of vermicompost form and dose within form were not significant (*p* > 0.05).

**Figure 6:**
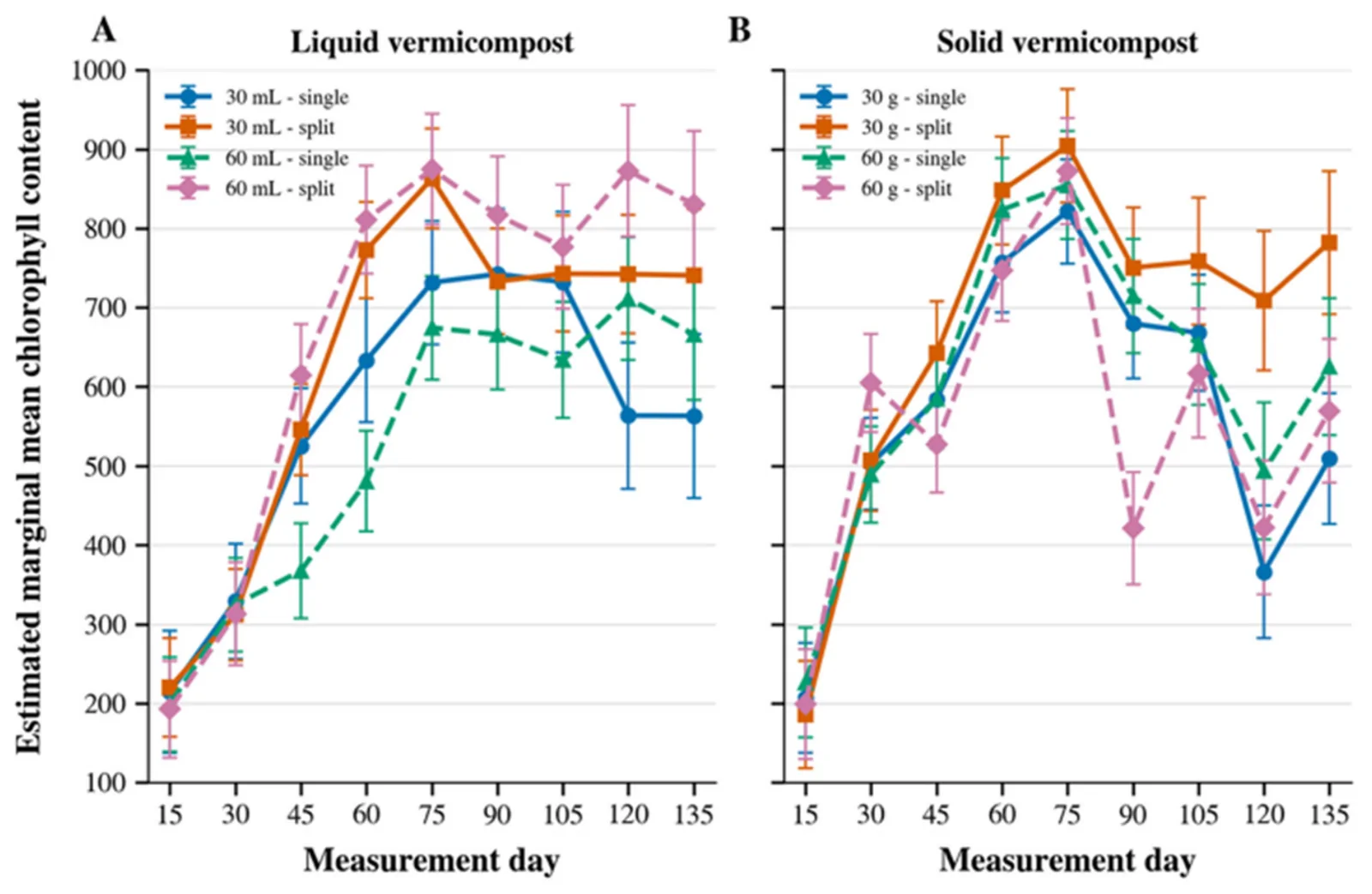
Dose within vermicompost form × application schedule × time interaction for leaf chlorophyll content. (**A**) liquid vermicompost treatments; (**B**) solid vermicompost treatments. Values are estimated marginal means with 95% confidence intervals.

As shown in Figure 6, estimated marginal means indicated that temporal changes in leaf chlorophyll content depended on dose within vermicompost form and application schedule. On day 135, the highest estimated chlorophyll content was obtained with 60 mL of liquid vermicompost applied in split doses (830.58 µmol m^−2^), followed by 30 g of solid vermicompost applied in split doses (782.27 µmol m^−2^). In contrast, 60 g of solid vermicompost applied in split doses showed marked declines on days 90 and 120. These contrasting temporal trajectories indicate that no single treatment combination consistently produced the highest leaf chlorophyll content across all measurement occasions.

## 4. Discussion

The growth responses observed in tea seedlings may reflect the combined roles of vermicompost as a nutrient source and soil amendment capable of influencing physical, chemical, and biological processes in the root zone. In particular, selected split-dose solid vermicompost treatments produced the greatest descriptive increases in seedling height and root collar diameter. These responses may be associated with the biochemical composition of solid vermicompost and its capacity to sustain nutrient availability in the root zone [31]. Humic substances, plant growth regulators, and beneficial microorganisms contained in vermicompost have been reported to promote root development, improve nutrient uptake efficiency, and consequently support increases in seedling height and root collar diameter [32,33,34]. Although root development was not measured in the present study, enhanced early growth may contribute to the establishment of a stronger seedling structure. However, whether this advantage persists during subsequent growth stages requires longer-term evaluation.

The changes observed in pH and electrical conductivity following vermicompost treatments suggest that vermicompost may influence the chemical properties of the soil. The results of the two-way analysis of variance revealed that the measurement time × treatment interaction significantly affected pH, EC, and soil temperature, indicating that temporal changes in these variables differed among treatments. Tea (*Camellia sinensis*) prefers slightly acidic soil conditions for optimal growth and nutrient uptake. The pH values recorded in this study (4.90–5.76) remained within the general range considered suitable for tea cultivation, suggesting that the treatments did not shift soil pH to levels likely to restrict seedling growth [35].

The modestly higher mean pH values recorded in the solid vermicompost treatments may indicate their potential to moderate growing-medium acidification under high-rainfall conditions. Similarly, the higher EC values observed in some treatments may reflect an increase in soluble ions, including plant nutrients. However, because all EC values remained within a low range, the growing medium was unlikely to have imposed substantial salinity stress on the seedlings. This pattern may be consistent with the gradual release of nutrients from vermicompost, although nutrient-release dynamics were not measured directly in this study. Vermicompost applications have also been reported to modify soil reaction, stimulate microbial activity, and promote plant growth by improving nutrient availability [35,36,37,38,39].

The findings of this study are generally consistent with previous reports that vermicompost applications promote seedling development [19,20,21,40,41,42,43]. In the present study, selected solid vermicompost treatments produced more pronounced increases in seedling height and root collar diameter than the liquid vermicompost treatments. However, this apparent advantage was primarily associated with morphological growth, because the leaf chlorophyll responses depended on the application schedule and its interactions with vermicompost form and dose within form rather than on the main effect of form alone. The stronger morphological responses to solid vermicompost may be associated with differences in the retention and availability of organic matter, nutrients, and microbial components in the root zone [44]. In contrast, liquid vermicompost may provide more rapidly available nutrients and bioactive compounds, although their persistence in the growing medium may be shorter. Under the high-rainfall conditions of the study area, differences in nutrient retention and leaching may also have contributed to the contrasting responses to solid and liquid vermicompost; however, nutrient losses were not measured directly. This interpretation is consistent with previous studies describing prolonged nutrient availability from solid vermicompost and faster but potentially shorter-term physiological responses to liquid vermicompost products [14,16,32,33,34,45,46].

Dividing the same total fertilizer amount among multiple applications was associated with greater final seedling height and root collar diameter in some treatment combinations, particularly under solid vermicompost. However, the REML results showed that the effects of application schedule depended on vermicompost form, dose within form, measurement time, and the trait evaluated; therefore, split application was not consistently superior across all treatments and measurement occasions. One possible explanation is that split applications may have synchronized nutrient supply more closely with plant demand and reduced potential nutrient losses compared with a single application. However, because nutrient losses and fertilizer-use efficiency were not measured directly, this explanation remains hypothetical. The findings nevertheless indicate that application timing should be considered together with vermicompost form and dose in organic fertilizer management. Heavy rainfall and excessive irrigation have been reported to increase nutrient losses and reduce fertilizer-use efficiency [47,48,49,50,51]. Similarly, fertilizer application timing has been reported to play an important role in growth performance and nutrient use efficiency in *Eucalyptus grandis* × *E. urophylla* seedlings [52]. More broadly, recent tea propagation studies have shown that early plantlet growth responses can vary considerably depending on treatment conditions and the growing medium [53].

The higher final chlorophyll contents observed in some split-dose treatment combinations suggest that application schedule may influence temporal chlorophyll responses. Leaf chlorophyll content exhibited a different response pattern from seedling height and root collar diameter. Leaf chlorophyll responses may also vary with measurement conditions and timing, as chlorophyll-related optical responses can be influenced by diurnal thermal and light conditions [54]. Although selected solid vermicompost treatments produced greater increases in morphological traits, the main effect of vermicompost form on chlorophyll content was not significant. Instead, chlorophyll dynamics depended on application schedule and its interactions with vermicompost form, dose within form, and measurement time. The highest final adjusted chlorophyll content was obtained with 60 mL liquid vermicompost applied in split doses, whereas solid vermicompost treatments produced higher values at some other measurement occasions. Therefore, the results do not demonstrate an overall physiological superiority of either vermicompost form. Measurement of leaf N concentration could help determine whether the observed increases in chlorophyll content were associated with improved nitrogen nutritional status in the seedlings. Because leaf nutrient concentrations and photosynthetic activity were not measured, the contrasting chlorophyll trajectories cannot be attributed directly to differences in nutrient availability, nutrient-use efficiency, or photosynthetic performance.

Liquid vermicompost applications have also been reported to increase leaf chlorophyll content and improve plant physiological performance [27,28,29,55]. Similarly, vermicompost applications during the seedling stage have been reported to increase chlorophyll content and promote the accumulation of other photosynthetic pigments, including carotenoids [56,57,58]. These findings provide relevant context for the chlorophyll responses observed in the present study, although their magnitude and temporal pattern varied according to vermicompost form, dose within form, application schedule, and measurement time.

Overall, the supplementary treatment-wise comparisons indicated that split applications provided advantages in some treatment combinations. However, the factorial longitudinal analyses showed that the effects of application schedule were trait- and time-dependent and could not be characterized solely by the main effects of application schedule or dose. Vermicompost form and its interaction with time significantly affected seedling height and root collar diameter, whereas leaf chlorophyll content was influenced by application schedule, time, and several interactions involving vermicompost form and dose within form (Table 10). Because significant higher-order interactions were detected for all three traits, the corresponding main effects and lower-order interactions should be interpreted in the context of these higher-order interactions. Under the environmental conditions of this study, split applications may therefore offer advantages for certain vermicompost form–dose combinations, but their superiority should not be generalized across all traits and measurement periods. These findings underscore the importance of considering vermicompost form, dose within form, application schedule, and measurement time jointly when developing vermicompost management strategies for tea seedlings.

## 5. Conclusions and Recommendations

In this study, the effects of vermicompost applications on seedling height, root collar diameter, leaf chlorophyll content, and selected physicochemical properties of the growing medium were evaluated over time in *Camellia sinensis* seedlings. The findings showed that seedling growth and chlorophyll responses varied among treatments and measurement periods, with these temporal patterns influenced by vermicompost form, dose within form, application schedule, and their interactions.

Supplementary treatment-wise comparisons and baseline-adjusted estimated marginal means showed that specific split applications of solid vermicompost produced the greatest final seedling height and root collar diameter values. However, the mixed-model results indicated that these responses depended on measurement time and treatment combination and therefore did not represent a uniform advantage of split application across all conditions. The observed advantages of split nutrient supply in certain combinations may be associated with more sustained nutrient availability, although this mechanism was not directly measured in the present study.

Leaf chlorophyll content varied significantly with application schedule and measurement time, together with several interactions involving vermicompost form and dose within form. However, the overall main effect of vermicompost form was not significant. Although certain liquid vermicompost treatments produced large relative increases in chlorophyll content, solid vermicompost treatments reached higher absolute chlorophyll values during several measurement periods, demonstrating that the response depended on the specific treatment combination and measurement time. Because leaf nutrient concentrations, photosynthetic rate, and pigment composition were not measured, the physiological mechanisms underlying these responses could not be determined directly.

Vermicompost treatment, measurement time, and their interaction significantly affected soil pH, electrical conductivity, and temperature. Nevertheless, the observed pH values remained within the acidic range considered suitable for tea cultivation, while the EC values remained low and were unlikely to impose substantial salinity stress under the conditions of this study. These findings suggest that the tested vermicompost treatments modified the physicochemical conditions of the growing medium without shifting its pH or EC beyond ranges considered suitable for tea seedlings during the experimental period.

Overall, dose within vermicompost form did not have a significant main effect on seedling height, root collar diameter, or leaf chlorophyll content in the factorial mixed models. Application schedule significantly affected the root collar diameter and chlorophyll content but not seedling height, while significant higher-order interactions were detected for all three traits. Therefore, the influence of application schedule cannot be interpreted independently of vermicompost form, dose within form, measurement time, and the response trait evaluated. Under the environmental conditions of this study, split applications appear promising for selected vermicompost form–dose combinations; however, their superiority should not be generalized across all traits and measurement periods. This study provides longitudinal evidence that application schedule and temporal response should be considered jointly when developing vermicompost management practices for tea seedlings.

### Recommendations

- Under the conditions of this study, selected split applications of solid vermicompost can be considered promising for improving seedling height and root collar diameter. However, their effectiveness should be validated across different growing media, tea genotypes, environmental conditions, and longer-term field trials before broader practical recommendations are made.
- Certain liquid vermicompost treatments produced substantial relative increases in leaf chlorophyll content and may warrant further evaluation for their effects on physiological performance. However, these responses should not be interpreted as direct evidence of improved plant nutrition because leaf nutrient concentrations and photosynthetic performance were not measured.
- In a combined fertilization strategy, solid vermicompost may be applied in split doses as the primary root-zone nutrient source, while liquid vermicompost may be used as a supplementary root-zone application during active seedling growth
- Future studies should jointly assess leaf and growing-medium nutrient contents, root development, microbial activity, photosynthetic performance, and nutrient losses through leaching to clarify the mechanisms underlying the observed responses.
- The potential use of vermicompost as an environmentally sustainable organic fertilizer source in tea cultivation should be evaluated through economic analyses and producer-scale trials.

## Figures and Tables

**Table 1:** Some physicochemical properties of solid and liquid vermicompost.

Parameters	Solid Vermicompost	Liquid Vermicompost
N (%)	2.53	0.94
P_2_O_5_ (mg kg^−1^)	1748.25	1104
K (mg kg^−1^)	7790	2875.23
Ca (mg kg^−1^)	12,942.75	4526.77
Mn (mg kg^−1^)	1626.50	156.51
S (mg kg^−1^)	1478.50	469.44
Al (mg kg^−1^)	6599.75	4522.54
pH	6.36	5.81
EC (μS/cm)	437.75	864.25
Moisture (%)	42.56	NA
Organic Matter (%)	32.29	NA

**Table 2:** Vermicompost applications used in the study and experimental design.

Treatment	Vermicompost Form	Total Dose	Application Mode	Management Groups	Seedlings per Management Group	Initial Number of Seedlings (n)
T0	Control	-	-	6	5	30
T1	Liquid	30 mL	Single application	6	5	30
T2	Liquid	30 mL	Split application (10 mL × 3)	6	5	30
T3	Liquid	60 mL	Single application	6	5	30
T4	Liquid	60 mL	Split application (20 mL × 3)	6	5	30
T5	Solid	30 g	Single application	6	5	30
T6	Solid	30 g	Split application (10 g × 3)	6	5	30
T7	Solid	60 g	Single application	6	5	30
T8	Solid	60 g	Split application (20 g × 3)	6	5	30

**Table 3:** Root collar diameter of tea seedlings under different vermicompost treatments at each measurement occasion, with the results of one-way ANOVA and Tukey’s HSD test. SD = standard deviation. Within each measurement day, means followed by different lowercase letters are significantly different according to Tukey’s HSD test (*p* < 0.05), whereas means sharing the same letter are not significantly different.

Days		Treatments									F (*p*)
		T0	T1	T2	T3	T4	T5	T6	T7	T8	
Day 0	Mean	6.88	7.12	6.69	6.63	6.57	7.17	7.30	6.95	6.96	0.903 (0.515)
	S.D.	1.29	1.05	1.29	1.24	0.98	1.40	1.08	1.33	1.26	
	Group	a	a	a	a	a	a	a	a	a	
Day 15	Mean	7.64	7.65	7.62	7.15	7.81	8.00	8.10	7.50	7.78	1.011 (0.429)
	S.D.	1.35	1.17	1.44	1.32	1.25	1.38	1.02	1.47	1.38	
	Group	a	a	a	a	a	a	a	a	a	
Day 30	Mean	8.18	8.35	7.81	7.67	8.10	9.54	9.35	8.23	8.91	5.463 (<0.001)
	S.D.	1.48	1.41	1.33	1.27	1.22	1.32	1.53	1.41	1.24	
	Group	ab	ab	a	a	ab	c	c	ab	bc	
Day 45	Mean	9.04	9.24	8.58	8.50	9.48	11.38	11.60	11.45	11.52	16.524 (<0.001)
	S.D.	1.50	1.75	1.42	1.32	1.44	1.84	2.14	1.82	1.83	
	Group	ab	ab	a	a	a	b	b	b	bc	
Day 60	Mean	9.14	9.24	8.65	8.63	9.81	11.58	11.90	11.73	12.39	21.288 (<0.001)
	S.D.	1.53	1.75	1.47	1.31	1.69	1.72	1.92	1.70	1.88	
	Group	ab	ab	a	a	b	c	c	c	c	
Day 75	Mean	9.29	9.29	8.73	8.96	10.07	11.71	12.55	11.73	12.52	20.872 (<0.001)
	S.D.	1.63	1.72	1.46	1.17	1.79	1.90	2.09	1.70	1.86	
	Group	ab	ab	a	a	b	c	c	c	c	
Day 90	Mean	9.53	9.35	9.00	9.38	10.62	11.58	13.05	12.00	12.70	19.051 (<0.001)
	S.D.	1.61	1.77	1.60	1.21	1.94	1.86	2.35	1.60	1.99	
	Group	a	a	a	a	b	bc	d	cd	d	
Day 105	Mean	9.88	9.53	9.27	9.71	10.76	11.75	13.55	12.09	13.13	17.465 (<0.001)
	S.D.	1.69	1.74	1.59	1.30	1.97	1.82	3.02	1.66	2.01	
	Group	ab	a	a	ab	bc	cd	f	de	ef	
Day 120	Mean	9.94	9.53	9.31	9.75	10.76	12.21	13.75	12.18	13.39	18.317 (<0.001)
	S.D.	1.72	1.74	1.64	1.36	1.97	2.11	3.11	1.65	2.10	
	Group	ab	a	a	ab	b	c	d	c	d	
Day 135	Mean	10.39	9.76	9.46	9.92	10.81	12.75	14.00	12.55	13.96	17.772 (<0.001)
	S.D.	1.99	1.79	1.70	1.56	2.06	2.35	3.15	1.71	2.16	
	Group	ab	a	ab	ab	b	cd	d	c	d	

**Table 4:** Mean seedling height, standard deviations, and results of one-way ANOVA and Tukey’s HSD test for tea seedlings under different vermicompost treatments at each measurement occasion. SD = standard deviation. Within each measurement day, means followed by different lowercase letters are significantly different according to Tukey’s HSD test (*p* < 0.05), whereas means sharing the same letter are not significantly different.

Days		Treatments									F (*p*)
		T0	T1	T2	T3	T4	T5	T6	T7	T8	
Day 0	Mean	12.02	12.18	12.08	12.29	12.29	12.83	11.80	11.77	12.48	0.910 (0.509)
	S.D.	1.42	1.47	1.81	1.78	1.52	2.12	1.99	1.72	1.50	
	Group	a	a	a	a	a	a	a	a	a	
Day 15	Mean	11.84	12.79	12.25	12.90	12.95	12.33	12.50	11.41	12.65	1.849 (0.069)
	S.D.	1.42	1.67	1.97	2.25	1.52	2.48	2.09	2.13	1.64	
	Group	a	a	a	a	a	a	a	a	a	
Day 30	Mean	12.78	13.18	12.88	14.33	14.00	13.88	13.50	12.32	13.48	2.431 (0.015)
	S.D.	1.58	1.33	2.27	2.32	1.90	2.42	2.09	2.79	2.02	
	Group	ab	abc	ab	c	bc	bc	abc	a	abc	
Day 45	Mean	14.81	14.53	13.77	14.60	15.38	19.21	18.75	18.00	19.26	13.565 (<0.001)
	S.D.	2.07	1.62	2.77	2.13	2.13	3.66	4.36	3.73	4.32	
	Group	a	a	a	a	a	b	b	b	b	
Day 60	Mean	15.13	15.41	15.27	15.92	16.24	20.71	19.55	19.59	20.74	14.899 (<0.001)
	S.D.	2.15	2.00	2.59	2.72	2.17	4.12	3.90	3.46	5.22	
	Group	a	a	a	a	a	b	b	b	b	
Day 75	Mean	15.63	15.82	14.90	16.96	17.50	19.75	21.40	19.05	21.96	16.055 (<0.001)
	S.D.	2.26	2.14	2.55	2.39	1.48	3.76	4.35	3.11	5.56	
	Group	ab	abc	a	b	bc	de	ef	cd	f	
Day 90	Mean	17.02	16.44	16.38	18.13	17.57	22.38	23.65	20.86	23.91	17.117 (<0.001)
	S.D.	2.92	1.89	2.40	2.24	1.58	4.57	4.94	3.69	6.54	
	Group	a	a	a	a	a	bc	c	b	c	
Day 105	Mean	17.26	16.79	16.83	18.04	18.12	22.67	24.00	20.55	23.30	15.116 (<0.001)
	S.D.	3.02	2.33	2.38	2.69	1.97	4.31	4.60	3.76	6.48	
	Group	a	a	a	a	a	bc	c	b	c	
Day 120	Mean	16.39	15.53	15.77	16.25	16.19	23.04	24.05	21.00	23.00	22.638 (<0.001)
	S.D.	2.91	2.24	2.86	2.56	1.99	4.37	4.84	3.32	6.60	
	Group	a	a	a	a	a	bc	c	b	bc	
Day 135	Mean	16.96	16.47	16.79	17.46	17.33	22.75	24.80	21.18	24.26	20.715 (<0.001)
	S.D.	2.69	2.04	2.81	2.39	1.89	4.48	4.44	3.57	5.97	
	Group	a	a	a	a	a	bc	c	b	c	

**Table 5:** Absolute and percentage changes in seedling height between day 0 and day 135 under different vermicompost treatments.

Parameters	Treatments								
	T0	T1	T2	T3	T4	T5	T6	T7	T8
Day 0 (cm)	12.02	12.18	12.08	12.29	12.29	12.83	11.80	11.77	12.48
Day 135 (cm)	16.96	16.47	16.79	17.46	17.33	22.75	24.80	21.18	24.26
Amount of change (cm)	4.94	4.29	4.71	5.17	5.04	9.92	13.00	9.41	11.78
Rate of change (%)	41.10	35.22	38.99	42.07	41.01	77.32	110.17	79.95	94.39

Note: Rate of change (%) = [(Day 135 − Day 0)/Day 0] × 100. Treatment codes are defined in Table 2.

**Table 6:** Mean leaf chlorophyll content, standard deviations, and results of one-way ANOVA and Tukey’s HSD test under different vermicompost treatments at each measurement occasion. SD = standard deviation. Within each measurement day, means followed by different lowercase letters are significantly different according to Tukey’s HSD test (*p* < 0.05), whereas means sharing the same letter are not significantly different.

Days		Treatments									F (*p*)
		T0	T1	T2	T3	T4	T5	T6	T7	T8	
Day 15	Mean	216.15	198.91	220.36	197.30	192.49	206.58	180.50	235.00	213.41	0.904 (0.514)
	S.D.	94.79	50.61	113.95	45.91	62.41	63.59	62.73	66.56	66.40	
	Group	a	a	a	a	a	a	a	a	a	
Day 30	Mean	334.96	299.49	330.69	316.22	301.67	520.34	482.94	486.16	604.11	7.977 (<0.001)
	S.D.	133.16	149.78	126.39	81.02	138.44	250.76	264.21	255.11	285.47	
	Group	a	a	a	a	a	b	b	b	b	
Day 45	Mean	460.54	513.54	551.33	368.18	614.80	579.07	619.28	584.73	527.45	8.547 (<0.001)
	S.D.	152.58	155.57	144.19	83.06	123.96	107.89	139.81	118.97	219.50	
	Group	b	bc	cd	a	d	cd	d	cd	bcd	
Day 60	Mean	741.74	655.42	779.31	491.28	810.12	750.98	861.95	830.19	747.17	12.45 (<0.001)
	S.D.	107.56	173.88	182.82	143.12	144.67	153.01	105.09	161.78	195.89	
	Group	bc	b	cd	a	cd	bc	d	cd	bcd	
Day 75	Mean	787.80	726.61	867.16	679.05	874.46	818.99	914.98	848.23	872.40	7.658 (<0.001)
	S.D.	138.04	168.08	138.43	104.11	94.25	137.32	139.65	92.21	185.61	
	Group	bc	ab	cd	a	cd	c	d	cd	cd	
Day 90	Mean	725.95	738.71	732.48	674.42	816.76	684.55	755.30	726.25	421.05	7.712 (<0.001)
	S.D.	206.02	176.50	224.40	184.81	103.89	223.06	199.87	192.83	150.57	
	Group	bc	bc	bc	b	c	b	bc	bc	a	
Day 105	Mean	698.42	744.19	717.44	633.60	776.55	667.90	758.52	653.33	620.54	1.658 *p* = 0.111
	S.D.	207.64	125.14	185.99	181.25	108.02	246.15	210.53	221.51	218.29	
	Group	a	a	a	a	a	a	a	a	a	
Day 120	Mean	616.96	560.26	751.70	719.45	872.48	346.11	702.50	465.17	398.53	18.917 (<0.001)
	S.D.	201.64	216.58	217.19	176.04	109.25	123.67	165.82	155.34	224.26	
	Group	de	cd	f	ef	g	a	ef	bc	ab	
Day 135	Mean	581.79	539.71	733.92	673.22	825.35	528.32	738.78	636.55	557.15	5.940 (<0.001)
	S.D.	209.37	204.41	200.07	170.61	97.04	243.92	211.49	189.85	166.46	
	Group	ab	a	cd	bc	d	a	cd	abc	ab	

**Table 7:** Absolute and percentage changes in leaf chlorophyll content between days 15 and 135 under different vermicompost treatments.

Parameters	T0	T1	T2	T3	T4	T5	T6	T7	T8
**Day 15 (Mean ± SD)**	216.15 ± 94.79	198.91 ± 50.61	220.36 ± 113.95	197.30 ± 45.91	192.49 ± 62.41	206.58 ± 63.59	180.50 ± 62.73	235.00 ± 66.56	213.41 ± 66.40
**Day 135 (Mean ± SD)**	581.79 ± 209.37	539.71 ± 204.41	733.92 ± 200.07	673.22 ± 170.61	825.35 ± 97.04	528.32 ± 243.92	738.78 ± 211.49	636.55 ± 189.85	557.15 ± 166.46
**Amount of change (µmol m^−2^)**	365.64	340.80	513.56	475.92	632.86	321.74	558.28	401.55	343.74
**Rate of change (%)**	169.16	171.33	233.06	241.22	328.78	155.75	309.30	170.87	161.07

Note: Rate of change (%) = [(Day 135 − Day 15)/Day 15] × 100. Treatment codes are defined in Table 2.

**Table 8:** Two-way ANOVA results for the effects of measurement time, vermicompost treatment, and their interaction on growing-medium properties.

Variable	Source	Type III Sum of Squares	df	Mean Square	F	*p*-Value
pH	Measurement time	18.34	8.00	2.29	41.56	<0.001
	Treatment	8.73	8.00	1.09	19.79	<0.001
	Measurement time × treatment	14.55	64.00	0.23	4.12	<0.001
Soil Temperature (°C)	Measurement time	18,066.58	8.00	2258.32	1023.10	<0.001
	Treatment	114.45	8.00	14.31	6.48	<0.001
	Measurement time × treatment	441.57	64.00	6.90	3.13	<0.001
EC (mS cm^−1^)	Measurement time	0.32	8.00	0.04	15.58	<0.001
	Treatment	0.17	8.00	0.02	8.29	<0.001
	Measurement time × treatment	0.31	64.00	0.00	1.87	0.001

**Table 9:** Marginal means, standard deviations, and multiple-comparison groups for growing-medium pH, temperature, and electrical conductivity (mS cm^−1^) according to measurement time and vermicompost treatment. SD = standard deviation. Within each measurement day, means followed by different lowercase letters are significantly different according to Tukey’s HSD test (*p* < 0.05), whereas means sharing the same letter are not significantly different.

Variables		Day 15	Day 30	Day 45	Day 60	Day 75	Day 90	Day 105	Day 120	Day 135
pH	Mean	5.10	5.02	5.76	5.08	4.90	5.04	5.47	5.13	5.03
	S.D.	0.23	0.26	0.47	0.37	0.34	0.27	0.64	0.19	0.20
	Group	b	ab	d	b	a	b	cd	b	ab
Soil T. (°C)	Mean	25.49	29.99	29.35	32.75	28.57	27.64	16.72	11.19	8.48
	S.D.	2.36	0.79	0.97	3.36	1.26	1.90	2.30	1.88	1.06
	Group	d	g	g	h	f	e	c	b	a
EC (mS cm^−1^)	Mean	0.19	0.18	0.15	0.08	0.22	0.16	0.16	0.17	0.17
	S.D.	0.09	0.07	0.05	0.04	0.08	0.05	0.05	0.05	0.04
	Group	d	cd	b	a	e	bcd	bc	bcd	bcd
**Variables**		**T0**	**T1**	**T2**	**T3**	**T4**	**T5**	**T6**	**T7**	**T8**
pH	Mean	5.21	5.00	4.96	4.97	4.93	5.38	5.40	5.39	5.27
	S.D.	0.31	0.27	0.41	0.36	0.29	0.51	0.46	0.55	0.45
	Group	b	a	a	a	a	c	c	c	bc
Soil T. (°C)	Mean	22.49	24.32	24.08	23.27	23.89	22.77	22.81	23.45	23.95
	S.D.	8.71	9.76	8.77	8.43	8.71	8.59	8.33	8.47	8.57
	Group	a	d	cd	abc	cd	ab	ab	bc	cd
EC (mS cm^−1^)	Mean	0.13	0.17	0.15	0.15	0.15	0.21	0.18	0.18	0.19
	S.D.	0.05	0.04	0.05	0.05	0.04	0.10	0.09	0.09	0.07
	Group	a	bc	ab	ab	ab	d	cd	cd	cd

Note. Soil T = soil temperature (°C); EC = electrical conductivity (mS cm^−1^); SD = standard deviation. Within each row, means followed by the same lowercase letter are not significantly different according to Tukey’s HSD test at *p* ≤ 0.05. Because the measurement time × treatment interaction was significant for all three variables, the marginal means and Tukey groupings are presented for descriptive purposes and should be interpreted together with the time-specific treatment patterns shown in Figure 1, Figure 2 and Figure 3.

**Table 10:** Type III tests of the experimental fixed effects from the linear mixed-effects models fitted by restricted maximum likelihood (REML) for seedling height, root collar diameter, and leaf chlorophyll content.

Fixed Effect	Seedling Height				Root Collar Diameter				Leaf Chlorophyll Content			
	df_1_	df_2_	*F*	*p*	df_1_	df_2_	*F*	*p*	df_1_	df_2_	*F*	*p*
Vermicompost form	1	165.57	103.30	**<0.001**	1	164.88	105.62	**<0.001**	1	166.43	0.03	0.868
Dose within form	1	165.77	2.79	0.097	1	163.10	2.02	0.158	1	166.43	2.26	0.135
Application schedule	1	165.64	3.29	0.072	1	163.23	7.35	**0.007**	1	166.43	19.74	**<0.001**
Form × application schedule	1	165.59	3.56	0.061	1	163.48	0.40	0.529	1	166.43	7.04	**0.009**
Dose within form × application schedule	1	166.63	0.04	0.841	1	163.36	1.35	0.246	1	166.43	15.23	**<0.001**
Time	8	811.95	193.40	**<0.001**	8	784.70	155.71	**<0.001**	8	912.30	239.76	**<0.001**
Form × time	8	811.95	45.69	**<0.001**	8	784.70	31.94	**<0.001**	8	912.30	28.98	**<0.001**
Dose within form × time	8	811.95	1.71	0.092	8	784.70	1.83	0.068	8	912.30	3.86	**<0.001**
Application schedule × time	8	811.95	1.76	0.081	8	784.70	1.89	0.059	8	912.30	7.87	**<0.001**
Form × application schedule × time	8	811.95	2.91	**0.003**	8	784.70	0.78	0.624	8	912.30	4.69	**<0.001**
Dose within form × application schedule × time	8	811.95	0.82	0.589	8	784.70	2.04	**0.039**	8	912.30	4.73	**<0.001**

Note. df_1_ = numerator degrees of freedom; df_2_ = denominator degrees of freedom. Dose was nested within vermicompost form. The models for seedling height and root collar diameter were adjusted for the corresponding day-0 values. No baseline adjustment was made for leaf chlorophyll content because it was not measured on day 0. Significant *p* values (*p* < 0.05) are shown in bold.

## Data Availability

The original contributions presented in the study are included in the article. Further inquiries can be directed to the corresponding author.

## Abbreviations

The following abbreviations are used in this manuscript:

ANOVA	Analysis of Variance
CRD	Completely Randomized Design
EC	Electrical Conductivity
HSD	Honestly Significant Difference
IBM	International Business Machines
NA	Not Available
REML	Restricted Maximum Likelihood
SD	Standard Deviation
SPSS	Statistical Package for the Social Sciences
USD	United States Dollar
